# Supplementary material for: A network medicine approach to investigating ME/CFS pathogenesis in severely ill patients: a pilot study
Source: Front Hum Neurosci. 2025 Feb 10;19:1509346. doi: 10.3389/fnhum.2025.1509346 (PMC11847890; doi:10.3389/fnhum.2025.1509346)
Supplement: Supplementary file 2 [file Data_Sheet_1.docx]

## A network medicine approach on investigating ME/CFS pathogenesis in severely ill patients: a pilot study

### Supplementary Figures


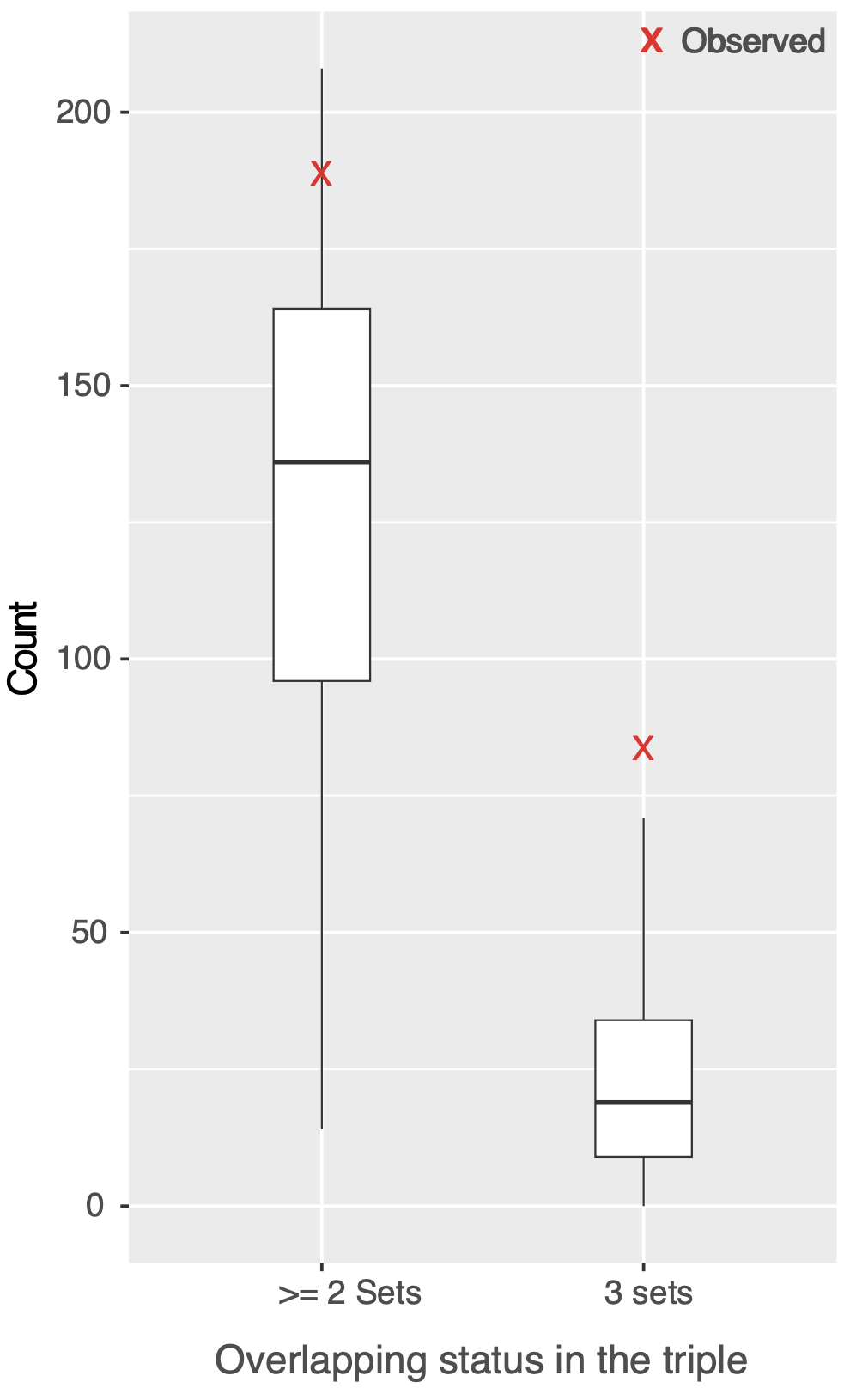


Supplementary Figure 1. Simulation results showing the overlapping status of the three ME/CFS disease modules. We generated 1000 randomly shuffled seed sets for each disease module. The Y-axis represents the number of overlapping nodes, while the X-axis represents the number of sets in which the nodes overlap.

###
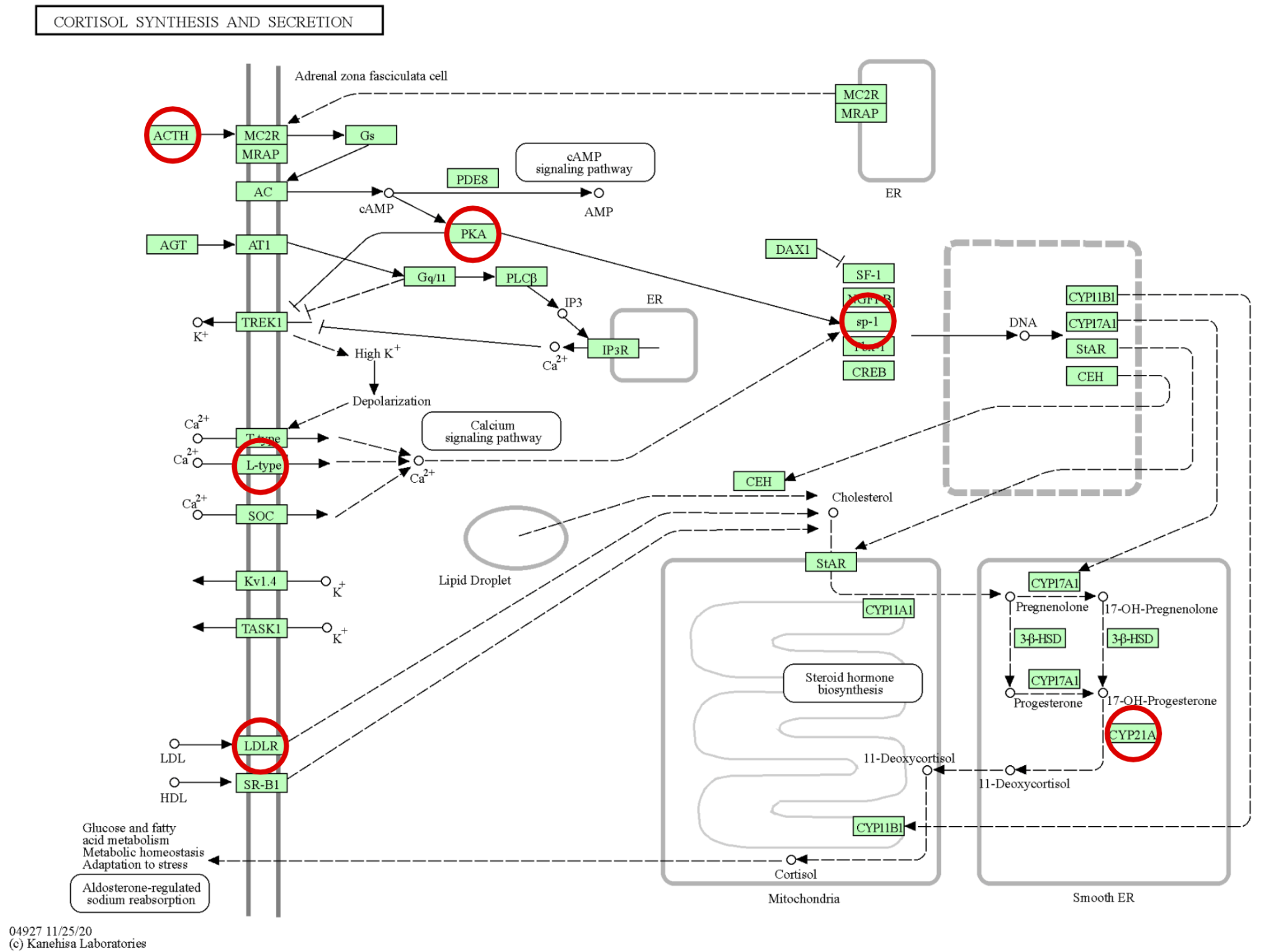


Supplementary Figure 2. The overlap between ME/CFS disease modules and the pathway of cortisol synthesis and secretion. Genes in the overlap are circled in red. The pathway and its graph were obtained from KEGG (https://www.kegg.jp).
